# Supplementary material for: The ecological drivers of variation in global language diversity
Source: Nat Commun. 2019 May 3;10:2047. doi: 10.1038/s41467-019-09842-2 (PMC6499821; doi:10.1038/s41467-019-09842-2)
Supplement: Supplementary file 1 — Supplementary Information [file 41467_2019_9842_MOESM1_ESM.pdf]

Supplementary information for:

The ecological drivers of global variation in language diversity

Hua et al.

Contents:

Supplementary Figure 1. Phylogenetic similarity in language diversity among grid cells under high resolution.
Supplementary Figure 2. Scatter plots of language diversity against each of the six climatic variables and four landscape variables.
Supplementary Figure 3. Global distribution of vascular plant diversity and amphibian diversity under high resolution.
Supplementary Figure 4. Scatter plots of language diversity against vascular plant diversity, amphibian diversity, mammal diversity, and bird diversity.
Supplementary Figure 5. Scatter plot of residual language diversity against the number of language families in each grid cell.
Supplementary Figure 6. Grid sub-sampling strategy for the global distribution of language diversity.

Supplementary Table 1. Continental specific landscape effects on language diversity.
Supplementary Table 2. Association between biodiversity and residual language diversity that cannot be explained by the climatic and landscape variables.
Supplementary Table 3. Climatic effects on language diversity at high resolution under alterative subsampling.
Supplementary Table 4. Landscape effects on language diversity and speaker population size after accounting for their covariation with climatic variables at high resolution under alternative subsampling.

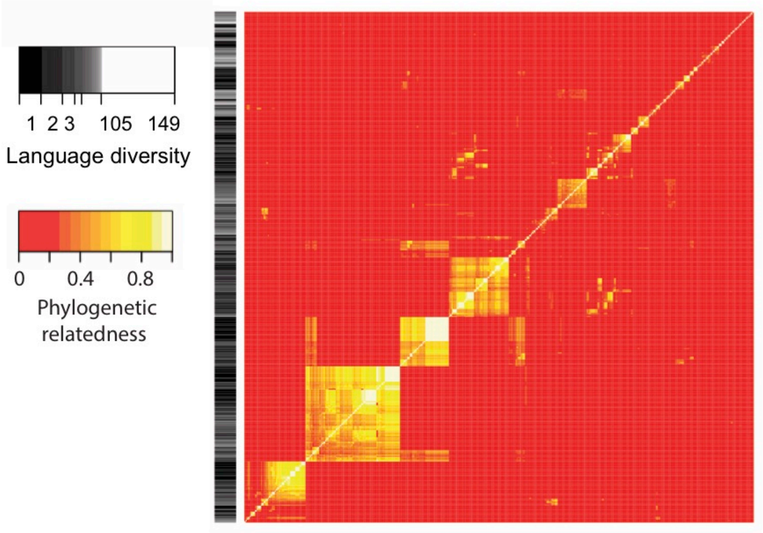

Supplementary Figure 1: Phylogenetic similarity matrix in language diversity among grid cells under high resolution. Each row and each column corresponds to a grid cell. Between each pair of grid cells, phylogenetic similarity due to relatedness of languages was calculated using the PhyloSor metric (see Methods), colored in a red to yellow scale with increasing phylogenetic relatedness. Grid cells with high phylogenetic relatedness are clustered together, with those having the same languages colored in white. For example, the largest white block refers to English and the second largest white block refers to Spanish. Language diversity in each row grid cell is colored in a black to white scale with increasing diversity, plotted as each stripe on the left bar to the matrix. Grid cells with high phylogenetic relatedness (the white-yellow blocks) tend to have low language diversity (grey-black stripes). Highly related languages in these grid cells with low language diversity may generate incidental associations between some feature of these languages and language diversity, if phylogenetic relatedness is not accounted for. Language data from WLMS 16 worldgeodatasets.com.

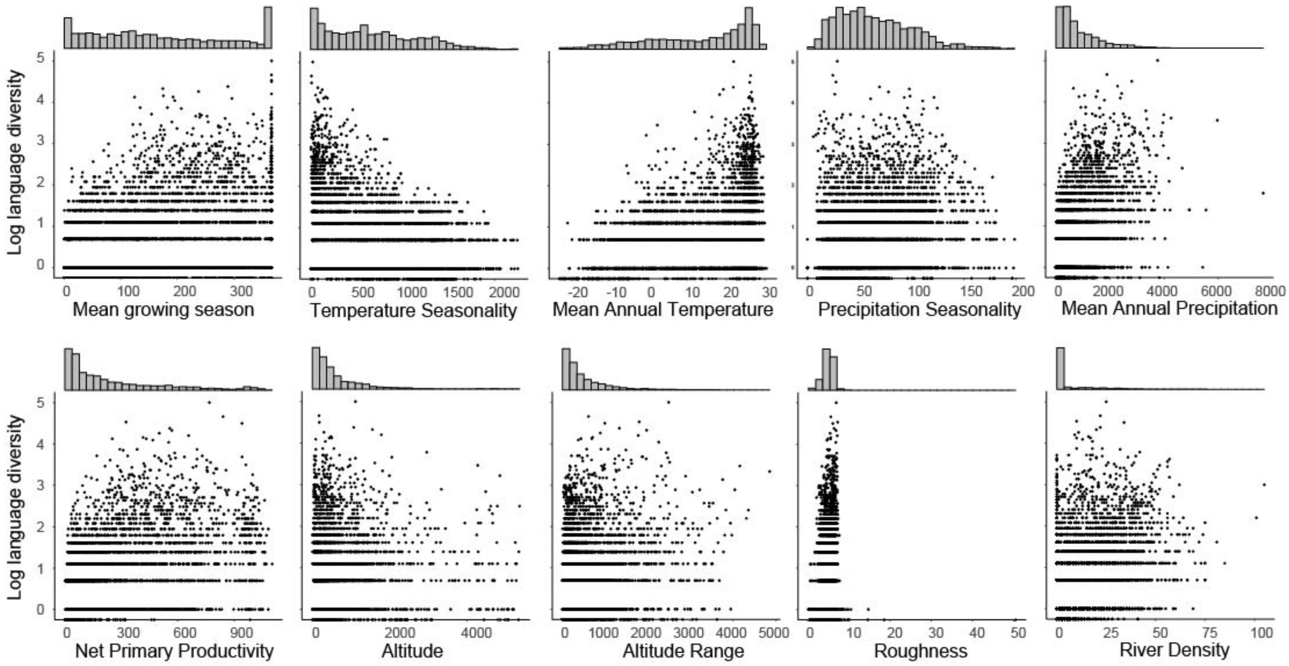

Supplementary Figure 2. Scatter plots of language diversity against each of the six climatic variables and four landscape variables. Each data point is a grid cell under high resolution without subsampling. Histogram of each climatic and landscape variable per grid cell is plotted along the corresponding axis. Language data from WLMS 16 worldgeodatasets.com.

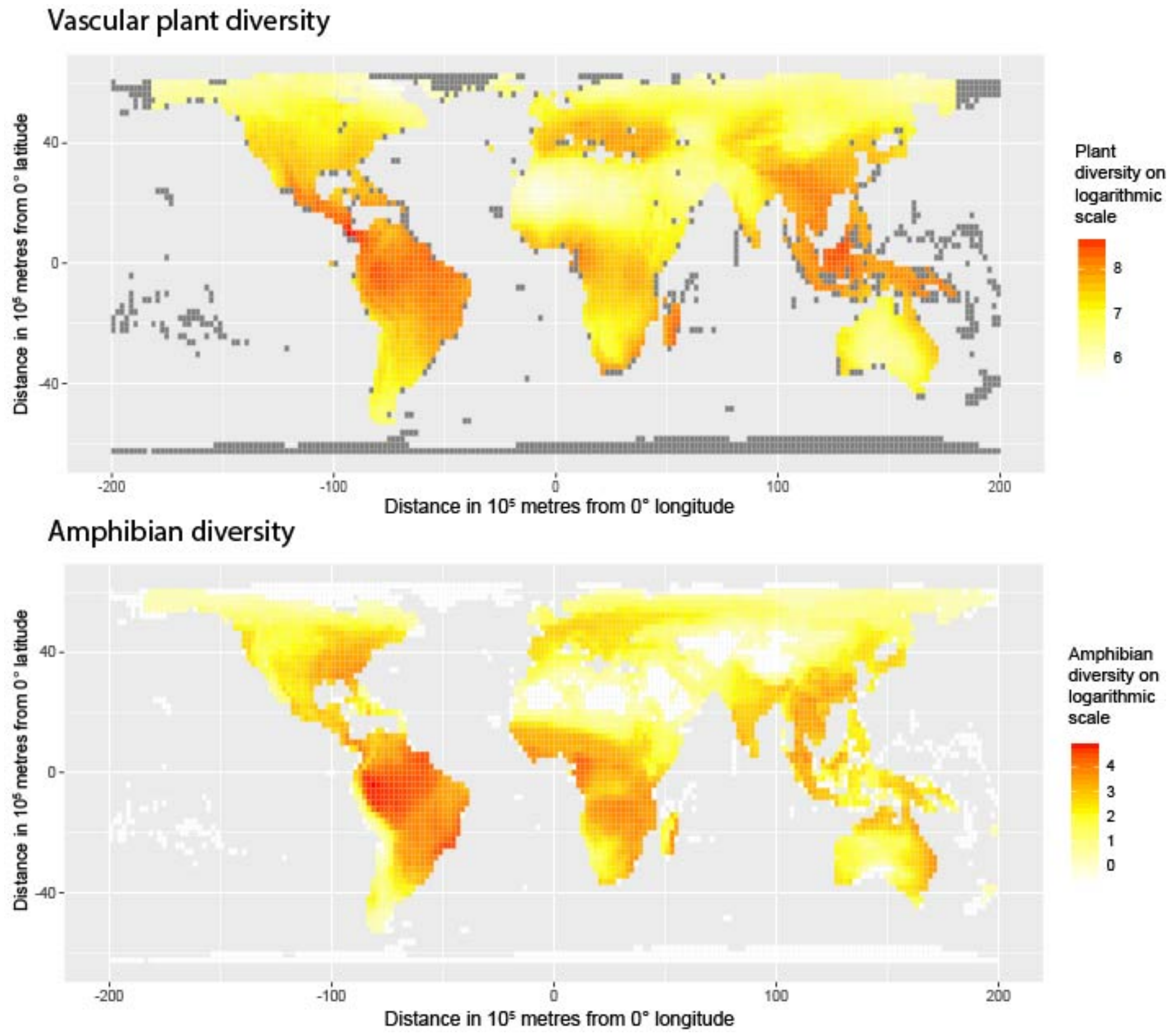

Supplementary Figure 3. Global distribution of vascular plant diversity and amphibian diversity. Values on logarithm scale of number of species are shown for 200x200km cells of an equal-area grid.

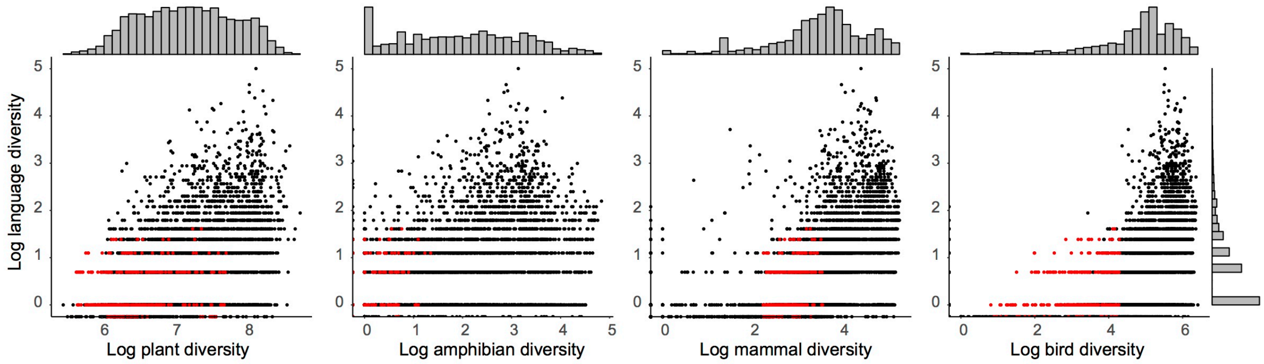

Supplementary Figure 4. Scatter plots of language diversity against vascular plant diversity, amphibian diversity, mammal diversity, and bird diversity. Each data point is a grid cell under high resolution without subsampling. Histogram of each of our four biodiversity measures per grid cell is plotted along the corresponding axis. Grid cells that fall in the Sahara, the Arabian Peninsula, and the Tibetan Plateau are plotted in red. These are the regions that all the four species groups have low diversity due to the harsh environments, which contribute to the positive association between language diversity and biodiversity (especially bird diversity). Language data from WLMS 16 worldgeodatasets.com.

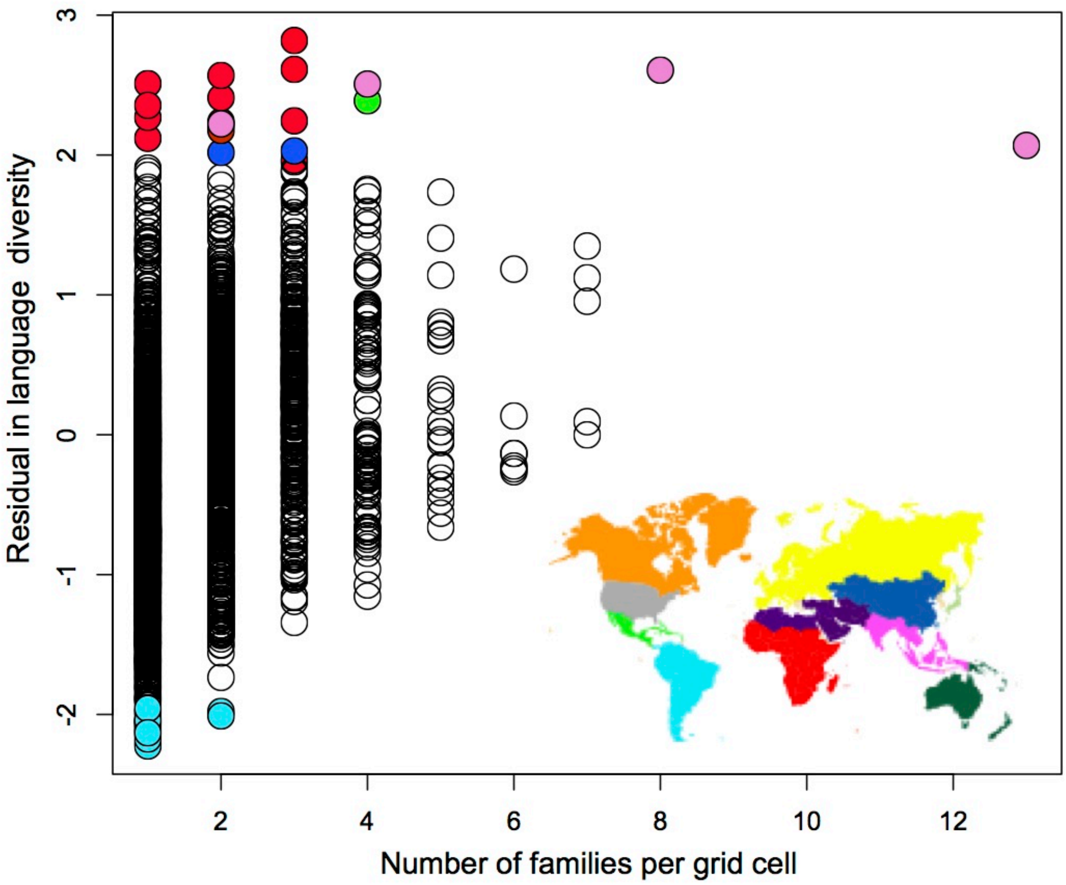

Supplementary Figure 5. Scatter plot of residual language diversity against the number of language families in each grid cell. Grid cells with residuals ≥ 1.96 or ≤ -1.96 are colored according to the regions (defined in Figure 1) at which they are located. The number of language families is a significant predictor for residual language diversity after correcting for spatial autocorrelation and phylogenetic relatedness (see main text). Language data from WLMS 16 worldgeodatasets.com.

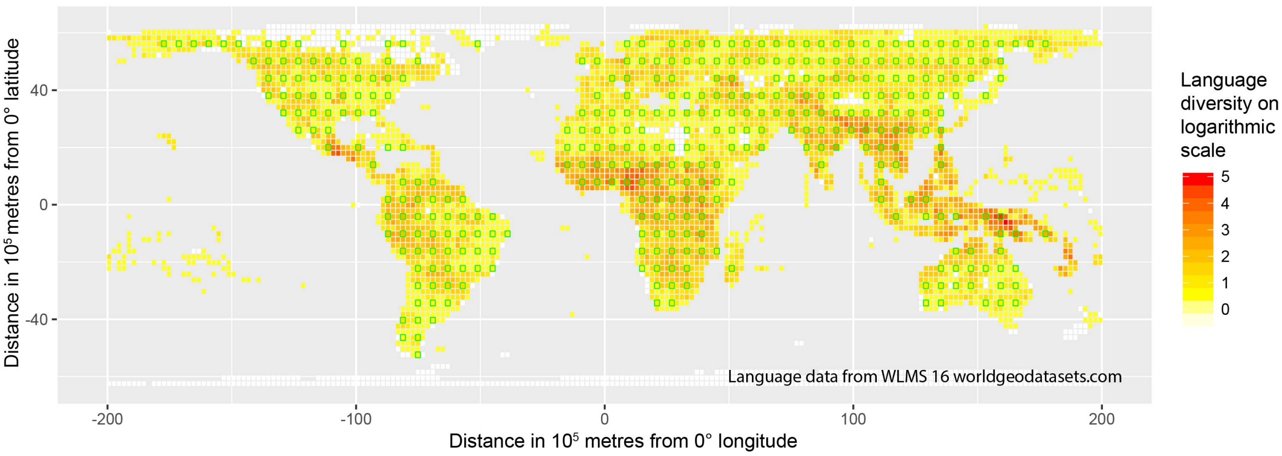

Supplementary Figure 6: Grid sub-sampling strategy for the global distribution of language diversity (number of languages per grid cell). To produce informative residual correlation matrix, green-bordered grids were sampled by removing their surrounding highly correlated grid cells in the analyses (See Methods for details).

Supplementary Table 1. Continental specific landscape effects on language diversity.

Method	Predictor	Africa		Europe		Extended Asia		Americas	
		t	*p*	*t*	*p*	*t*	*p*	*t*	*p*
AM GLS	Average altitude	0.35	0.724	0.46	0.648	-0.27	0.785	-0.49	0.625
	Landscape roughness	0.67	0.505	1.02	0.308	1.19	0.236	-0.16	0.875
	River density	1.29	0.197	-0.96	0.340	-0.08	0.936	-1.05	0.295
AM OLS	Average altitude	**3.39**	**<0.001**	0.19	0.851	**-3.14**	**0.002**	-0.73	0.463
	Landscape roughness	0.87	0.383	**2.11**	**0.035**	**3.97**	**<0.001**	**2.18**	**0.029**
	River density	**5.56**	**<0.001**	**-3.38**	**0.001**	-1.59	0.112	-1.73	0.083
ISO OLS	Average altitude	**3.33**	**0.001**	-1.21	0.228	-1.21	0.225	-0.92	0.357
	Landscape roughness	-0.35	0.728	1.56	0.119	**5.85**	**<0.001**	**3.07**	**0.002**
	River density	**3.11**	**0.002**	**-2.42**	**0.016**	1.21	0.225	-0.24	0.807

We list the *t* value and the *p* value of each landscape variable in a regression model that includes all the climatic and landscape variables under high resolution. Following the method of Axelson & Manrubia (2014), ordinary least squares (OLS) was used for regression without correcting for spatial autocorrelation and phylogenetic relatedness. Generalized least squares (GLS) was used for regression with correction. We used two ways to set the boundaries between continents: AM using the boundaries defined by Axelsen and Manrubia (2014)[18]; ISO using the boundaries between adjacent countries that belong to different continents, according to the associated continent of ISO3 country codes. Significant results are in bold. Extended Asia includes Asia and Oceania. Americas include North America and South America. Africa is the reference category, so the *t* value for other continents shows the effect of each variable on language diversity relative to the effect in Africa.

Supplementary Table 2. Association between biodiversity and residual language diversity that cannot be explained by the climatic and landscape variables.

Biodiversity	Low (*n*=216)		Medium (*n*=192)		High (*n*=366)	
	t	*p*	*t*	*p*	*t*	*p*
Plant diversity	0.97	0.335	-0.29	0.775	0.28	0.781
Amphibian diversity	-0.458	0.561	-1.12	0.266	-0.13	0.894
Mammal diversity	**2.41**	**0.017**	0.43	0.667	1.49	0.136
Bird diversity	**2.89**	**0.004**	0.92	0.359	1.86	0.064

We list the *t* value and the *p* value in a generalized least squares regression that has the biodiversity variable as the predictor and residuals in language diversity as the response variable.

Supplementary Table 3. Climatic effects on language diversity at high resolution under alterative subsampling.

Predictor	*row1 col1*	*row1 col2*	*row1 col3*	*row2 col1*	*row2 col2*	*row2 col3*	*row3 col2*	*row3 col3*
Annual mean precipitation	1.11<br>(0.27)	**2.52**<br>**(0.01)**	**3.55**<br>**(0.00)**	1.12<br>(0.26)	0.23<br>(0.82)	1.55<br>(0.12)	1.87<br>(0.06)	1.78<br>(0.08)
Annual mean temperature	0.33<br>(0.74)	-0.60<br>(0.55)	1.48<br>(0.14)	0.69<br>(0.49)	1.08<br>(0.28)	0.42<br>(0.68)	-0.30<br>(0.76)	0.36<br>(0.72)
Precipitation seasonality	0.66<br>(0.51)	0.96<br>(0.34)	0.08<br>(0.94)	0.40<br>(0.69)	**2.16**<br>**(0.03)**	0.50<br>(0.62)	1.45<br>(0.15)	0.07<br>(0.95)
Temperature seasonality	**-2.22**<br>**(0.03)**	**-3.79**<br>**(0.00)**	**-2.66**<br>**(0.01)**	**-2.40**<br>**(0.02)**	**-2.35**<br>**(0.02)**	**-3.38**<br>**(0.00)**	**-3.23**<br>**(0.00)**	**-2.47**<br>**(0.01)**
Net primary productivity	1.29<br>(0.20)	-0.08<br>(0.94)	-1.73<br>(0.08)	0.44<br>(0.66)	1.00<br>(0.32)	0.39<br>(0.70)	0.47<br>(0.64)	1.03<br>(0.30)
Mean annual growing season	0.77<br>(0.44)	0.05<br>(0.96)	1.08<br>(0.28)	**2.33**<br>**(0.02)**	1.09<br>(0.28)	0.38<br>(0.71)	0.86<br>(0.39)	0.34<br>(0.73)

Different subsampling starts with different rows and columns. Under each subsampling, we list the t value and the p value (in parentheses) of each predictor in a generalized least squares regression that includes all the six eco-climatic predictors. Results are qualitatively the same as Table 1, in that temperature seasonality has consistently the strongest association with language diversity under high resolution.

Supplementary Table 4. Landscape effects on language diversity and speaker population size after accounting for their covariation with climatic variables at high resolution under alternative subsampling.

Response	Predictor	*row1 col1*	*row1 col2*	*row1 col3*	*row2 col1*	*row2 col2*	*row2 col3*	*row3 col2*	*row3 col3*
Language diversity	Average altitude	0.30	1.83	-1.03	-0.66	-0.37	-0.24	0.33	0.56
		(0.76)	(0.07)	(0.30)	(0.51)	(0.71)	(0.81)	(0.74)	(0.57)
	Altitudinal range	1.30	**1.99**	**2.03**	0.54	**2.91**	1.81	1.28	1.18
		(0.19)	**(0.05)**	**(0.04)**	(0.59)	**(0.00)**	(0.07)	(0.20)	(0.24)
	Landscape roughness	-0.09	0.29	-0.03	1.82	-0.24	-0.23	1.34	-0.12
		(0.93)	(0.77)	(0.97)	(0.07)	(0.73)	(0.82)	(0.18)	(0.90)
	River density	1.08	0.27	**2.02**	1.18	**2.79**	1.80	0.60	1.67
		(0.28)	(0.78)	**(0.04)**	(0.24)	**(0.01)**	(0.07)	(0.55)	(0.10)
Average speaker population size	Average altitude	0.32	-0.78	1.11	0.75	1.22	1.32	-0.14	0.58
		(0.75)	(0.44)	(0.27)	(0.46)	(0.22)	(0.19)	(0.89)	(0.56)
	Altitudinal range	-1.78	-1.94	-0.46	0.20	-1.35	**-2.53**	-0.76	-0.92
		(0.08)	(0.05)	(0.65)	(0.84)	(0.18)	**(0.01)**	(0.45)	(0.36)
	Landscape roughness	**2.85**	**2.41**	-0.09	0.00	0.96	**2.74**	0.94	1.04
		**(0.00)**	**(0.02)**	(0.93)	(1.00)	(0.34)	**(0.01)**	(0.35)	(0.30)
	River density	1.69	-0.84	0.25	0.01	-1.52	-0.17	-0.28	0.69
		(0.09)	(0.40)	(0.80)	(0.99)	(0.13)	(0.87)	(0.78)	(0.49)
Minimum speaker population size	Average altitude	1.03	-0.34	1.65	0.22	1.49	1.83	0.33	1.30
		(0.30)	(0.73)	(0.10)	(0.82)	(0.14)	(0.07)	(0.74)	(0.20)
	Altitudinal range	**-2.04**	**-2.10**	-1.33	-1.38	**-2.12**	**-2.66**	-1.71	-1.34
		**(0.04)**	**(0.04)**	(0.18)	(0.17)	**(0.03)**	**(0.01)**	(0.09)	(0.18)
	Landscape roughness	**2.63**	1.43	-0.01	1.61	1.20	**2.07**	1.91	0.71
		**(0.01)**	(0.15)	(0.99)	(0.11)	(0.23)	**(0.04)**	(0.06)	(0.48)
	River density	0.87	-1.58	0.11	0.04	**-2.16**	-1.01	-0.40	0.39
		(0.38)	(0.11)	(0.91)	(0.97)	**(0.03)**	(0.31)	(0.69)	(0.69)

Under each subsampling, we list the t value and the p value (in parentheses) of each landscape variable in a generalized least squares regression that includes all the six climatic and four landscape variables. Models with population size also control for population density. Results are qualitatively the same as Table 3. There are associations between language diversity and altitudinal range and between language diversity and river density. These associations do not support the hypothesis that landscape factors divide human populations into smaller groups, because larger altitudinal range and higher river density are more associated with smaller minimum speaker population size than smaller average speaker population size, and higher landscape roughness is associated with larger average speaker population size and larger minimum speaker population size, opposite to what would be expected if landscape roughness divided human populations into smaller groups.
