# Supplementary material for: The ecological drivers of variation in global language diversity
Source: Nat Commun. 2019 May 3;10:2047. doi: 10.1038/s41467-019-09842-2 (PMC6499821; doi:10.1038/s41467-019-09842-2)
Supplement: Supplementary file 2 — Reporting Summary [file 41467_2019_9842_MOESM2_ESM.pdf]

nature research

Corresponding author(s): Xia Hua

Last updated by author(s): Mar 13, 2019

Reporting Summary

Nature Research wishes to improve the reproducibility of the work that we publish. This form provides structure for consistency and transparency in reporting. For further information on Nature Research policies, see Authors & Referees and the Editorial Policy Checklist.

Statistics

For all statistical analyses, confirm that the following items are present in the figure legend, table legend, main text, or Methods section.

n/a	Confirmed	
☐	☒	The exact sample size (*n*) for each experimental group/condition, given as a discrete number and unit of measurement
☐	☒	A statement on whether measurements were taken from distinct samples or whether the same sample was measured repeatedly
☐	☒	The statistical test(s) used AND whether they are one- or two-sided<br>*Only common tests should be described solely by name; describe more complex techniques in the Methods section.*
☐	☒	A description of all covariates tested
☐	☒	A description of any assumptions or corrections, such as tests of normality and adjustment for multiple comparisons
☐	☒	A full description of the statistical parameters including central tendency (e.g. means) or other basic estimates (e.g. regression coefficient) AND variation (e.g. standard deviation) or associated estimates of uncertainty (e.g. confidence intervals)
☐	☒	For null hypothesis testing, the test statistic (e.g. *F*, *t*, *r*) with confidence intervals, effect sizes, degrees of freedom and *P* value noted<br>*Give P values as exact values whenever suitable.*
☒	☐	For Bayesian analysis, information on the choice of priors and Markov chain Monte Carlo settings
☐	☒	For hierarchical and complex designs, identification of the appropriate level for tests and full reporting of outcomes
☐	☒	Estimates of effect sizes (e.g. Cohen's *d*, Pearson's *r*), indicating how they were calculated

Our web collection on statistics for biologists contains articles on many of the points above.

Software and code

Policy information about availability of computer code

Data collection	All the data were collected from published or online databases using R, with no additional software used.
Data analysis	All the data analyses were performed using R packages described in the manuscript, with no additional software used.

For manuscripts utilizing custom algorithms or software that are central to the research but not yet described in published literature, software must be made available to editors/reviewers. We strongly encourage code deposition in a community repository (e.g. GitHub). See the Nature Research guidelines for submitting code & software for further information.

Data

Policy information about availability of data

All manuscripts must include a data availability statement. This statement should provide the following information, where applicable:

- Accession codes, unique identifiers, or web links for publicly available datasets
- A list of figures that have associated raw data
- A description of any restrictions on data availability

The language data that support the findings of the study are available from World Language Mapping System but restrictions apply to the availability of these data, which were used under license for the current study, and so are not publicly available. These data are however available from the authors upon reasonable request and with permission of World Language Mapping System. The other data are from published and publicly available databases (see text for details). Publications and web links for these datasets are reported in the references. Figure 1, 2, 4 and Supplementary Figure 2-4 have associated raw data.

Field-specific reporting

Please select the one below that is the best fit for your research. If you are not sure, read the appropriate sections before making your selection.

☐ Life sciences ☐ Behavioural & social sciences ☒ Ecological, evolutionary & environmental sciences

For a reference copy of the document with all sections, see nature.com/documents/nr-reporting-summary-flat.pdf

Ecological, evolutionary & environmental sciences study design

All studies must disclose on these points even when the disclosure is negative.

Study description	We present the first global analysis of language diversity that identifies the relative importance of two key ecological mechanisms suggested to promote language diversification - isolation and ecological risk - after correcting for spatial autocorrelation and phylogenetic non-independence.
Research sample	Our analyses were based on global-scale datasets of the geographic distribution of 6425 languages, high-resolution climatic and geographic data layers, and global biodiversity datasets. We used the number of languages whose distribution overlaps each cell of a global equal-area grid as the measure of language diversity.
Sampling strategy	All analyses were based on grid cells for a global equal-area projection at three different resolutions: low resolution with grid cell size 1000x1000km, medium resolution with grid cell size 500x500km, and high resolution with grid cell size 200x200km.
Data collection	All the data were from existing datasets.
Timing and spatial scale	The data were from the most-updated datasets. The scale is global.
Data exclusions	We excluded grid cells where no language distributions overlap that cell.
Reproducibility	We repeated analyses under three different spatial resolutions.
Randomization	Grid cells that are located near each other are likely to have similar values of climatic and landscape variables, contain related human cultures and languages, and share much of their flora and fauna. If there are any particular features of cultures or languages that correlate with language diversity, then they will tend to covary with environmental features and biodiversity measures, whether or not there is any causal connection between them. We accounted for this spatial autocorrelation and phylogenetic relatedness by constraining the residual correlation in language diversity between each pair of grid cells to be a linear function of the spatial proximity and phylogenetic similarity between the two cells.
Blinding	All the data were from existing datasets and the statistical analyses were standard, so blinding does not apply to this study.

Did the study involve field work? ☐ Yes ☒ No

Reporting for specific materials, systems and methods

We require information from authors about some types of materials, experimental systems and methods used in many studies. Here, indicate whether each material, system or method listed is relevant to your study. If you are not sure if a list item applies to your research, read the appropriate section before selecting a response.

Materials & experimental systems

n/a	Involved in the study	
☒	☐	Antibodies
☒	☐	Eukaryotic cell lines
☒	☐	Palaeontology
☒	☐	Animals and other organisms
☒	☐	Human research participants
☒	☐	Clinical data

Methods

n/a	Involved in the study	
☒	☐	ChIP-seq
☒	☐	Flow cytometry
☒	☐	MRI-based neuroimaging
